# C-Kit Promotes Growth and Migration of Human Cardiac Progenitor Cells via the PI3K-AKT and MEK-ERK Pathways

**DOI:** 10.1371/journal.pone.0140798

**Published:** 2015-10-16

**Authors:** Bathri N. Vajravelu, Kyung U. Hong, Tareq Al-Maqtari, Pengxiao Cao, Matthew C. L. Keith, Marcin Wysoczynski, John Zhao, Joseph B. Moore IV, Roberto Bolli

**Affiliations:** Institute of Molecular Cardiology, Department of Medicine, University of Louisville, Louisville, KY 40202, United States of America; Northwestern University, UNITED STATES

## Abstract

A recent phase I clinical trial (SCIPIO) has shown that autologous c-kit+ cardiac progenitor cells (CPCs) improve cardiac function and quality of life when transplanted into patients with ischemic heart disease. Although c-kit is widely used as a marker of resident CPCs, its role in the regulation of the cellular characteristics of CPCs remains unknown. We hypothesized that c-kit plays a role in the survival, growth, and migration of CPCs. To test this hypothesis, human CPCs were grown under stress conditions in the presence or absence of SCF, and the effects of SCF-mediated activation of c-kit on CPC survival/growth and migration were measured. SCF treatment led to a significant increase in cell survival and a reduction in cell death under serum depletion conditions. In addition, SCF significantly promoted CPC migration *in vitro*. Furthermore, the pro-survival and pro-migratory effects of SCF were augmented by c-kit overexpression and abrogated by c-kit inhibition with imatinib. Mechanistically, c-kit activation in CPCs led to activation of the PI3K and the MAPK pathways. With the use of specific inhibitors, we confirmed that the SCF/c-kit-dependent survival and chemotaxis of CPCs are dependent on both pathways. Taken together, our findings suggest that c-kit promotes the survival/growth and migration of human CPCs cultured *ex vivo* via the activation of PI3K and MAPK pathways. These results imply that the efficiency of CPC homing to the injury site as well as their survival after transplantation may be improved by modulating the activity of c-kit.

## Introduction

The adult mammalian heart has long been considered a post-mitotic organ that is incapable of regeneration. This notion was challenged by several reports suggesting that the heart is not terminally differentiated and is capable of regeneration, albeit to a limited extent [1–3]. Recent studies have shown that the mammalian heart harbors resident stem/progenitor cells that can contribute to tissue maintenance and repair. Several resident cardiac stem/progenitor cell (CPCs) populations have been reported in adult myocardium. These stem cell populations were initially identified based on their expression of common stem cell antigens, such as c-kit [4, 5] and Sca-1 [6, 7], or on their ability to efflux a fluorescent dye, Hoechst 33342 (“side population”) [8, 9] or to form spherical bodies (“cardiospheres”) under specific culture conditions [10, 11].

c-kit+ cells with properties of CPCs were first described in the rat heart by Beltrami et al. in 2003 [4]. When isolated and grown in culture, they were found to be self-renewing, clonogenic, and multipotent, being able to differentiate into cardiomyocytes, smooth muscle, and endothelial cells. Since then, c-kit+ CPCs with similar properties have been described in multiple mammalian species, including humans [5, 12–14]. The discovery of specialized niches which contain clusters of undifferentiated c-kit+ CPCs and early-lineage committed cells (i.e., c-kit and GATA4, MEF2C, or Ets1 double-positive cells) within the heart [13] strongly suggests that they not only reside stably in the heart but also are specifically “programmed” to give rise to multiple cardiac cell types. Moreover, when injected into an ischemic heart, c-kit+ CPCs have been reported to reconstitute myocardium with new vessels and myocytes [4]. In a recent phase I clinical trial (SCIPIO), c-kit+ CPCs isolated from patients with ischemic cardiomyopathy significantly improved cardiac function and structure as well as functional capacity and quality of life when transplanted back into the failing hearts via intracoronary injection [15], clearly demonstrating the utility of these cells in the treatment of ischemic heart diseases. Although several independent groups have highlighted the therapeutic benefits of c-kit+ CPCs, poor engraftment and limited survival of the transplanted cells remains one of the major hurdles [16–20]. In order to develop highly effective stem cell treatment, it is imperative to enhance some of the critical cellular characteristics of these cells, namely, survival, proliferation, migration and engraftment.

Cellular Kit (c-kit) (also known as CD117 or stem cell factor receptor) encodes a 145 kDa transmembrane glycoprotein that belongs to the type-III receptor tyrosine kinase family, which includes the platelet-derived growth factor receptor and macrophage colony stimulating factor 1 receptor [21–23]. Stem cell factor (SCF), also known as steel factor or kit ligand, is the only known ligand of c-kit. Ligand binding leads to dimerization of c-kit receptors followed by auto phosphorylation of tyrosine residues in its intracellular domain [24]. Activation of c-kit leads to recruitment and subsequent activation of a number of downstream mediators (e.g., Grb2, p85 subunit of PI3K, and PLCγ) [25–27]. Among these, phosphoinositol 3 kinase (PI3K) and the mitogen activated protein kinase (MAPK) pathways are critical pathways that are activated by c-kit/SCF [28–30]. c-Kit is a proto-oncogene and activating mutations in the c-kit gene are frequently associated with various types of tumors, such as mast cell tumors, gastrointestinal stromal tumors, and leukemia [31–33]. In addition to its role in tumorigenesis, studies in c-kit mutant mice have shown that it plays a critical role in regulating survival, proliferation, differentiation, and migration of various cell types, including mast cells [34, 35], melanocytes [36, 37], germ cells [38, 39] and vascular endothelial cells [40]. In addition, c-kit has been found to regulate the survival, maintenance, self-renewal, and homing of hematopoietic stem cells [41–44].

Although c-kit has been widely used as a surface antigen for identification and isolation of resident CPCs, its role in regulating the properties of CPCs remains unclear. In the present study, we hypothesized that c-kit plays a role in regulating the growth, survival, and migration of human c-kit+ CPCs. Our results indicate that activation of c-kit by SCF promotes the growth, survival and migration of human CPCs, and that these effects are mediated by the PI3K-AKT and MEK-ERK pathways.

## Materials and Methods

### Isolation and culture of c-kit+ human cardiac progenitor cells

Atrial appendages specimens were collected from patients during the coronary artery bypass graft performed at Jewish Hospital at University of Louisville. A written consent agreement was obtained for collection of discarded atrial appendages according to a protocol approved by the Institutional Review Board on human subject research at University of Louisville. c-kit+ human cardiac stem cells were isolated and cultured as previously described [45]. Written consent was obtained for collection of discarded atrial appendages according to a protocol approved by the Institutional Review Boards at University of Louisville. Briefly, atrial appendages obtained from patients undergoing coronary artery bypass surgery were enzymatically digested and cultured in complete growth medium (Ham’s F12 media containing 10% fetal bovine serum, 10 ng/ml bFGF [PeproTech], 0.005 U/ml human erythropoietin [Sigma], and 0.2 mM L-glutathione [Sigma]). Media was changed every two days. MACS kit (Miltenyl Biotec) was used to enrich for c-kit+/lin- CPCs following the manufacturer’s instructions as described previously [45]. Ham’s F12 media with 0.2 mM L-glutathione was used as assay medium for all the experiments unless mentioned otherwise.

### Cell growth and viability

Manual cell counting or PrestoBlue^TM^ (Invitrogen) was used to assess cell growth and viability. For manual cell counting, approximately 50,000 cells were plated per well of a 24-well plate. CPCs were then serum starved for 24 hr and treated with or without 100 ng/ml SCF in serum free media. For bFGF and VEGF treatments, CPCs were incubated with 50 ng/ml bFGF (Peprotech) or 20 ng/ml VEGF (Invitrogen) either alone or in combination with SCF as indicated. After 3 days of growth factor treatment, cells were trypsinized and counted using a hemacytometer. For measurement of cell viability using PrestoBlue^TM^, 10,000 CPCs were plated per well of a 96-well plate. When indicated, CPCs were treated with 0.5 μM imatinib mesylate (Cayman Chemicals) for 2 hr prior to SCF treatment. PrestoBlue^TM^ cell viability assay was performed 72 hr post-growth factor treatment according to the manufacturer’s instructions. Briefly, the 10x reagent was mixed with an appropriate volume of serum free medium, added to the cells and incubated at 37°C for an hr. The viability was assessed by measuring the fluorescence at Ex/Em 560/590 nm.

### BrdU assay

Approximately 10,000 cells per well were plated on a 96-well tissue culture plate. Cells were serum-starved for 24 hr followed by treatment with 20 μM BrdU for 24 or 72 hr in the presence or absence of SCF in the assay medium. After BrdU labeling, cells were fixed and immunostained using anti-BrdU antibody (at 1 in 1,000; Sigma-Aldrich #B8434) and Alexa555-conjugated secondary antibody (at 1 in 1,000; Invitrogen). Images were captured using EVOS® FL Cell Imaging System (Life Technologies), and the BrdU-positive cells were counted manually.

### Caspase assay

Apo-ONE^®^ Homogeneous Caspase-3/7 Assay kit (Promega) was used to measure the caspase activity in CPCs according to the manufacturer’s instructions. Briefly, approximately 10,000 CPCs were plated per well. The next day, the medium was changed to serum free media or same media containing SCF. After 3 days of SCF treatment, the caspase activity in the cells was measured by adding the pro-fluorescent substrate and reading the fluorescence at Ex/Em 499/521 nm. The values were normalized to the untreated control.

### Oxidative stress by DMNQ and H_2_O_2_

For DMNQ experiments, approximately 10,000 human c-kit+ CPCs per well were plated on a 96-well tissue culture plate. On the next day, cells were treated with 100 ng/ml SCF in serum free media. For the imatinib treatment groups, cells were treated with 0.5 μM imatinib for 2 hr prior to the SCF treatment. On the second day, CPCs were subjected to 8 μM DMNQ (dimethoxy-naphthoquinone; Sigma) treatment in the groups indicated. After 3 days of DMNQ treatment, cell viability was assessed using PrestoBlue^TM^. For H_2_O_2_ experiments, approximately 10,000 cells per well were plated on a 96-well tissue culture plate. On the next day, cells were treated with 100 ng/ml SCF or 10 μg/ml insulin (Gibco) in serum free media. On the following day, CPCs were subjected to 0.5 mM H_2_O_2_ (EMD Chemicals) treatment for 1 hr. Cell viability was assessed 3 days later using PrestoBlue^TM^.

### Western blot analysis

Cells were harvested with Laemlli buffer and heated for 10 minutes at 100°C. Protein concentration was estimated using bicinchoninic acid (BCA) assay kit purchased from Thermo scientific following manufacturer’s instructions. Before loading the samples on the gel, 3 μl of β-mercaptoethanol was added per 100 ul of sample and heated for 3 minutes at 100°C. Cell lysate (25 or 50 μg of protein) was loaded on 4–20% Tris-Glycine gel and separated by electrophoresis. The separated proteins were transferred to a PVDF membrane, blocked with 5% skim milk or 5% bovine serum albumin for 1 hr followed by probing with primary antibody overnight at 4°C. The blot was then incubated with HRP-conjugated secondary antibody for 1 hr and developed using Amersham ECL Prime Western Blotting Detection Reagent (GE Healthcare). Primary antibodies used were anti-c-kit (1 in 500; abcam #32363), anti-phospho c-kit (Y703; 1 in 500; Cell Signaling #3073P), anti-AKT (1in 1,000; Cell Signaling #4691), anti-phospho AKT (Thr 308; 1 in 1,000; Cell Signaling #13038), anti-ERK 1/2 (1in 1,000; Cell Signaling #4695P), anti-phospho ERK 1/2 (1 in 1,000; Cell Signaling #4370P), and anti-α-tubulin (1 in 3,000; Sigma #T6074).

### *In vitro* cell migration

Boyden chambers containing transwells (24-well plate format) with 8 μm pore size were purchased from Corning. Typically, 30,000–40,000 cells were plated in the upper chamber of the transwell. After 24 hr, the medium in the upper chamber was changed to assay medium and bottom chamber was filled with the same medium alone or medium containing 100 ng/ml SCF. For inhibition of c-kit activity, cells were pre-treated with 0.5 μM imatinib for 2 hr prior to the SCF treatment. After 24 hr, the cells migrated to the lower surface of the transwell membrane were fixed with 3.7% formaldehyde for 10 minutes, and the cells still remaining on the upper chamber were scraped off. The fixed cells were then permeabilized with 0.25% Triton X-100 in PBS for 10 minutes followed by incubation with 50 μg/ml propidium iodide solution for 10–15 minutes. Images were acquired using EVOS® FL Cell Imaging System (Life Technologies), and the number of migrated cells was quantified using the NIS-Elements (version 4.2) software.

### Lentivirus production and transduction

The lentivirus carrying mCherry or FLAG-tagged murine c-kit was produced by using the ViraPower^TM^ Lentiviral Expression System and pLenti6-V5 vector (Invitrogen) according to the manufacturer’s instructions. Virus was concentrated using the Lenti-X Concentrator (Clontech) and was stored at -80°C until its use. On the day of the experiment, CPCs were transduced with complete medium containing either mCherry or c-kit virus and 6 μg/ml polybrene (Sigma). On the following day, the media was replaced with fresh complete media. Cells were cultured for 5 days to allow expression of mCherry or c-kit prior to being used for the experiments.

### PI3K and MEK inhibitors

Wortmannin and PD98059 were purchased from Cell Signaling Technology and used at 200 nM and 40 μM concentrations, respectively. For the Western blot analysis, CPCs were serum starved for 24 hr and pre-treated with the inhibitors for 2 hr followed by 20 min of SCF (100 ng/ml) treatment. For the cell viability and migration assays, cells were pre-treated with the indicated inhibitors for 2 hr prior to being treated with SCF.

### Isolation and Differentiation of Murine c-kit+ CPCs

Mice (12–15 weeks old male C57BL6 mice) were euthanized by sodium pentobarbital injection (*i*.*p*. 100 mg/kg). The excised hearts were washed in PBS, minced into small pieces and enzymatically dissociated with Collagenase II (5 μg/ml in PBS) (Worthington) with gentle agitation for 45 min in 37°C. After Collagenase II inactivation with DMEM/F12 medium containing 10% FBS, cells were centrifuged at 600 xg for 10 min. The cell pellet was suspended in growth medium consisting of DMEM/F12, 10% FBS, bFGF (10 ng/ml), EGF (10 ng/ml), LIF (10 ng/ml), ITS (Insuline/Transferrine/Selenium), glutamine and penicillin/streptomycin. The single cell suspension was plated in the tissue culture flask. After 4–6 days of expansion, c-kit+ cells were sorted with using MACS immunomagnetic labeling method. Briefly, cells were detached by enzymatic digestion with 0.25% trypsin, washed in PBS and stained with rat monoclonal antibody anti-mouse c-kit (clone 2B8) conjugated with FITC (eBioscience) for 30 minutes on ice. Subsequently, cells were washed in PBS and stained with magnetic beads directed anti-FITC (Miltenyi) for 30 minutes on ice. The c-kit cells were sorted by positive selection with using MS magnetic column. The sorted cells were analyzed for the purity with using flow cytometry (LSRII, BD bioscience). On average 85% purity was obtained from all isolated fractions. Sorted cells with purity below 80% were excluded from the experiments. For differentiation, c-kit+ CPCs were passaged at seven days post-sort and seeded in on the 12-well plate at density of 50,000 cells per well. When cells reached approximately 90% of confluence, they were stimulated with differentiation or control media (DMEM, 5% FBS with or without dexamethasone [10 nM] accordingly) in the presence or absence of SCF (50 ng/ml). Cells were maintained for 4 weeks with media change once every two days. The myocyte, endothelial, and smooth muscle differentiation markers were evaluated at mRNA level by real time qPCR.

### Statistical analysis

Statistical analysis was performed by Mann-Whitney U test and Student’s t-test using SPSS ver. 17 software. Data are presented as mean ± SEM. P value of <0.05 was considered statistically significant.

## Results

### c-kit activation promotes growth and survival of human c-kit+ CPCs

**Fig 1:**
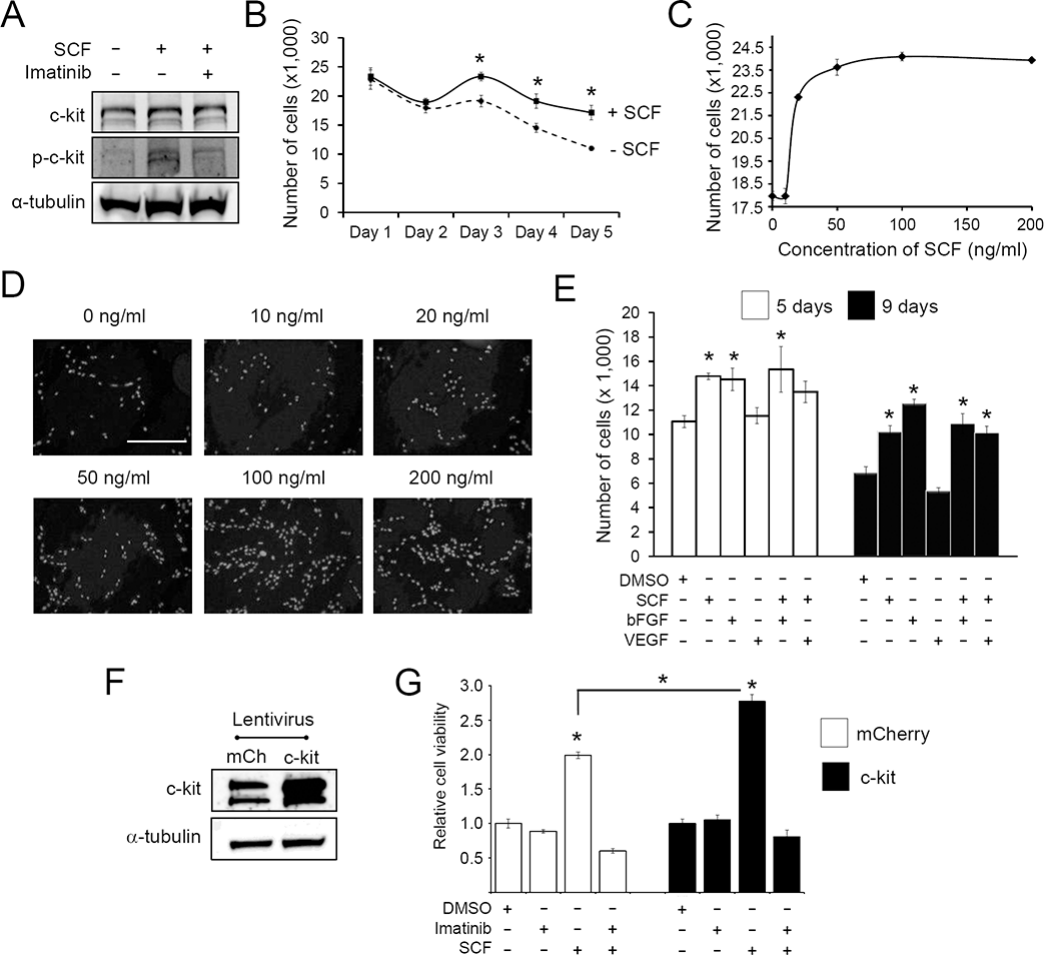
c-kit activation promotes growth of human c-kit+ CPCs under serum starvation. A, Activation of c-kit by SCF in human c-kit+ CPCs. CPCs were either treated with SCF (100 ng/ml) alone for 10 minutes or co-treated with imatinib. Cell lysate was analyzed by Western blot for the indicated proteins. p-c-kit, phosphorylated (i.e., activated) c-kit. B, Effect of SCF on cell growth. CPCs were serum starved for 24 hr and treated with SCF. The number of cells remaining was counted at the indicated time points. C, Dose-response relationship between SCF and CPC growth. CPCs were serum starved for 24 hr followed by treatment with varying concentrations of SCF, and the number of cells remaining after 3 days was determined. D, Representative DAPI nuclear staining images of SCF-treated CPCs described in panel C. E, Comparison of SCF with bFGF and VEGF in promoting growth of CPCs. CPCs were serum starved for 24 hr and treated with SCF, bFGF, or VEGF either individually or in combination, and the number of cells were counted on days 5 and 9 of treatment. F, Human c-kit+ CPCs were transduced with lentivirus expressing mCherry (control) or c-kit. Cell lysates were obtained at 4 days post-viral transduction and immunoblotted for c-kit and α-tubulin (loading control). G, mCherry or c-kit-expressing CPCs were serum starved for 24 hr and cultured for 3 days in the presence (+) or absence (-) of SCF and/or imatinib as indicated. Cell viability was assessed using PrestoBlue^TM^ as described. Values were normalized to the DMSO (vehicle) control. Values are presented as mean ± SEM. *, p<0.05. Scale bar represents 400 μm.

First, we tested if human CPCs express functional c-kit receptors. Western blot analysis confirmed expression of c-kit in CPCs, and treatment of CPCs with the c-kit ligand, SCF, increased the level of tyrosine phosphorylation of the c-kit receptor (Fig 1A). In addition, pre-treatment of the cells with the c-kit inhibitor imatinib abolished the SCF-induced phosphorylation of the receptor, as expected (Fig 1A). This indicates that human CPCs express functional c-kit receptors that can be activated by its ligand.

We then tested if SCF/c-kit signaling can act as a pro-growth or pro-survival pathway when CPCs are subjected to conditions of stress. For this purpose, we subjected CPCs to serum starvation. We tested if SCF could rescue the CPCs grown under serum starvation by treating them with or without SCF and measuring the cell number on each day until 5 days post-SCF treatment (Fig 1B). Compared to the vehicle control, SCF treatment resulted in a significantly greater number of cells starting at day 3 and up to day 5. Moreover, the pro-survival or pro-growth effect of SCF on CPCs was dose-dependent, and its effect appeared to plateau at 100 ng/ml (Fig 1C and 1D). Hence, 100 ng/ml concentration of SCF was chosen for all the subsequent experiments. Next, we compared the effect of SCF with those of other growth factors, including vascular endothelial growth factor (VEGF) and basic FGF (bFGF), in CPCs grown under serum starvation. At the same time, we also tested different combinations of growth factors. The pro-survival or–growth effect of SCF was comparable to that of bFGF in CPCs (Fig 1E). However, there was no additional or synergistic effect when cells were treated with both growth factors (Fig 1E), suggesting that they share similar downstream signaling pathways. To further confirm these findings, we overexpressed c-kit (or mCherry as a control) in human CPCs using lentivirus and subjected the cells to serum starvation in the presence or absence of SCF (Fig 1F). As expected, overexpression of c-kit resulted in a further increase in the number of cells when treated with SCF (Fig 1G). Such effect was abolished by pre-treatment of cells with imatinib, a c-kit inhibitor, which suggests that the effect of SCF on CPCs is indeed mediated through c-kit receptor (Fig 1G). These results indicate that SCF-mediated activation of c-kit enhances survival or growth of CPCs cultured under serum starvation.

### c-kit activation leads to increased proliferation of CPCs

**Fig 2:**
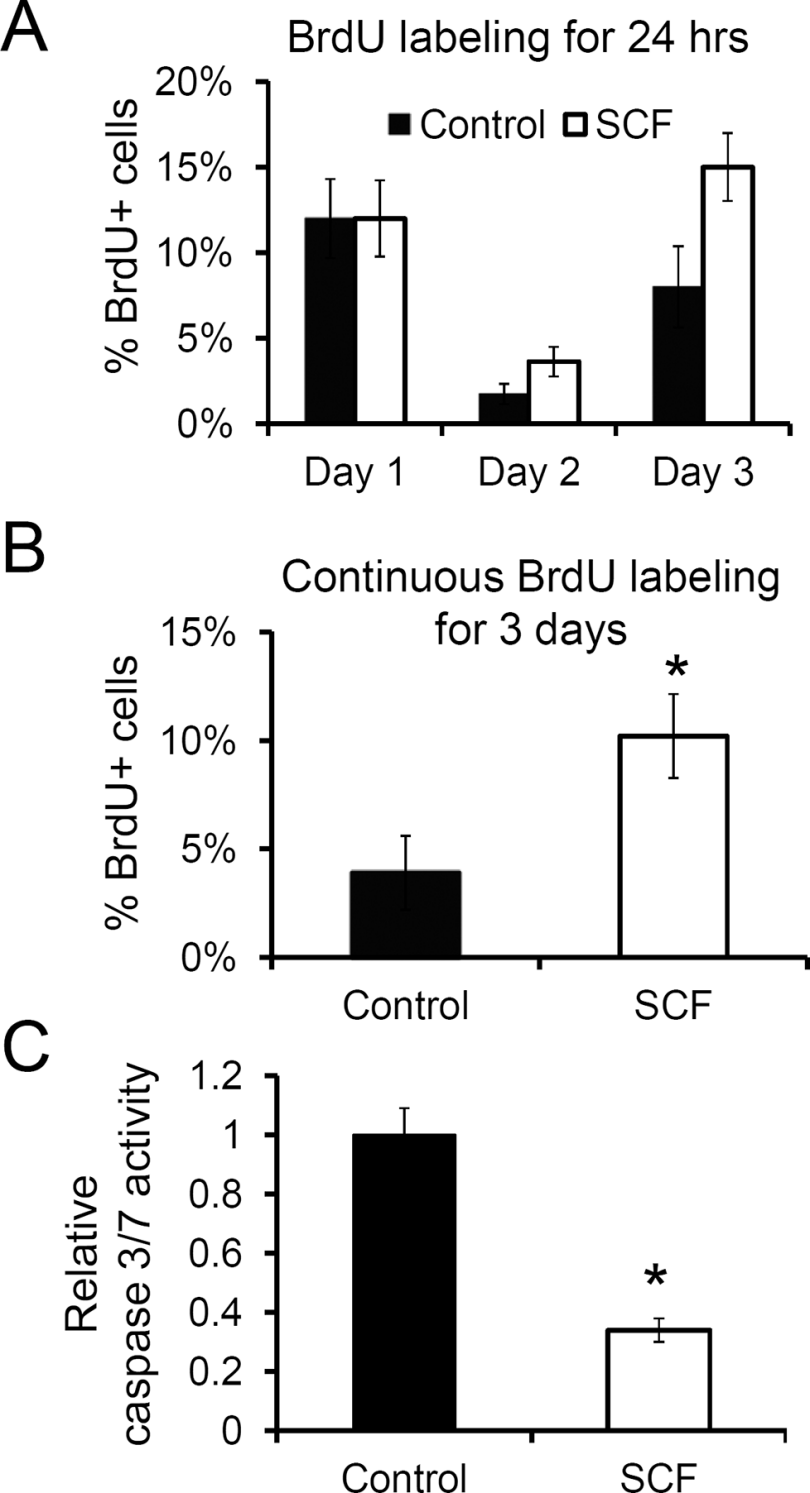
c-kit activation induces proliferation and decreases apoptotic cell death in CPCs. Human c-kit+ CPCs were serum starved for 24 hr and labeled with BrdU in the presence or absence of SCF for 24 hr only for the indicated day (A) or continuously for 3 days (B). Cells were stained with anti-BrdU antibody, and the positive cells were expressed as the percentage of total nuclei. C, CPCs were grown in serum free media for 3 days. Activities of caspases 3 and 7 were measured and normalized to the untreated control. Values presented are mean ± SEM. *, p<0.05.

In order to test whether the observed increase in the number of surviving cells was due to the mitogenic effect of SCF/c-kit signaling, we performed a BrdU labeling assay [46]. For this assay, we cultured the cells under serum starvation in the presence or absence of SCF and incubated them with BrdU for 24 hr starting at different time points. When compared to the control, SCF increased the BrdU labeling index in CPCs during days 2 and 3 of the treatment, suggesting that SCF treatment promotes proliferation in CPCs (Fig 2A). However, the difference between the two treatment groups did not reach statistical significance. Next, we treated the cells with BrdU continuously for 3 days with or without SCF. With continuous BrdU supply and SCF treatment, the percentage of BrdU-positive CPCs increased to 10.2 ± 3.9% (SCF-treated group) from 3.9 ± 3.4% (untreated control group), significantly raising the rate of proliferation by more than 2-fold (Fig 2B). This demonstrates that SCF acts as a mitogen for CPCs under conditions that restrict their growth.

### c-kit activation reduces apoptosis of CPCs under serum starvation

We then examined if SCF, besides acting as a mitogen, can also prevent apoptosis induced by serum depletion in CPCs. For this purpose, we measured the activation of caspases 3 and 7 in CPCs after culturing them in the presence or absence of SCF for 3 days. As shown in Fig 2C, SCF treatment resulted in a significant reduction in the caspase activity in these cells compared to the untreated control. Moreover, inhibiting c-kit with imatinib significantly diminished such anti-apoptotic effect of SCF in CPCs (data not shown). Taken together, these results indicate that c-kit activation by SCF produces mitogenic as well as anti-apoptotic effects in human CPCs.

### c-kit activation fails to rescue CPCs from oxidative stress

**Fig 3:**
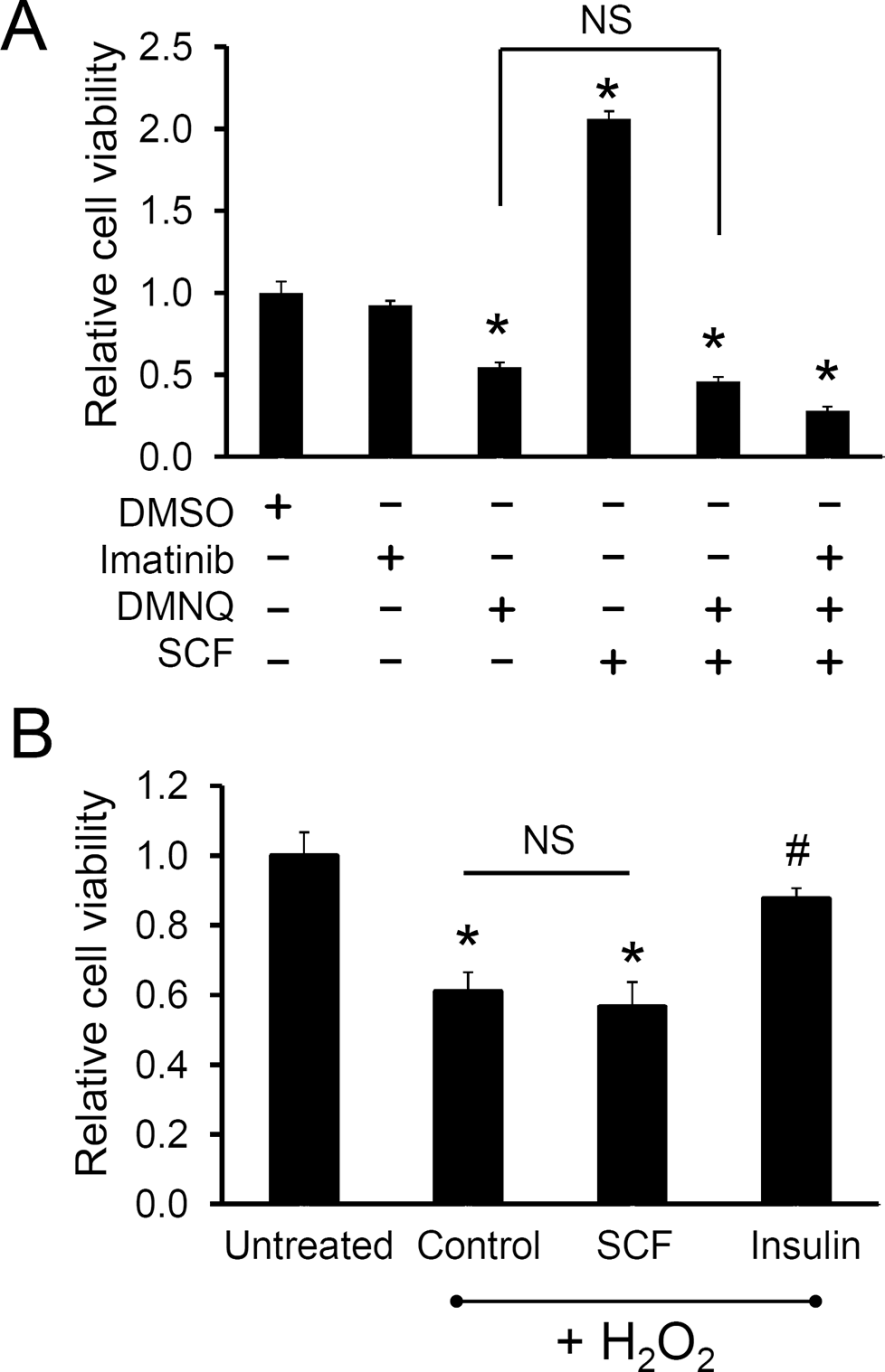
c-kit activation does not rescue CPCs from oxidative stress. A, Human c-kit+ CPCs were pre-treated with SCF for 24 hr in serum free media and subjected to 8 μM DMNQ treatment. B, Cells pre-treated with or without SCF received 1 hr treatment in 0.5 mM H_2_O_2_, followed by media change with or without SCF. Insulin was used as a positive control. Cell viability was assessed after 3 days using PrestoBlue^TM^. *, p<0.05 compared to the untreated control, #, p<0.05 compared to the H_2_O_2_ only control. NS, no statistical significance. Values presented are mean ± SEM. *, p<0.05.

Next, we tested if SCF can prevent cell death induced by other stress conditions, such as oxidative stress, in CPCs. For this, CPCs were pre-treated with SCF for 24 hr followed by treatment with an oxidative stress inducer, dimethoxy-naphthoquinone (DMNQ) or H_2_O_2_. Of note, DMNQ is a quinone that induces superoxide anion formation by redox cycling [47], whereas H_2_O_2_ undergoes the Fenton reaction in the presence of iron and produces reactive oxygen species, causing cell injury and death [48]. As shown in Fig 3A, DMNQ treatment led to a significant decrease in the number of cells. However, pre-treating the cells with SCF failed to prevent the cell death induced by DMNQ treatment (Fig 3A). Similarly, SCF treatment of CPCs was not able to prevent or attenuate the cell death following H_2_O_2_-induced oxidative stress (Fig 3B). In contrast, insulin pre-treatment (a positive control) was able to rescue CPCs from H_2_O_2_-induced oxidative stress (Fig 3C). These results indicate that while SCF-induced activation of c-kit promotes survival of CPCs grown under serum depletion, it does not recue CPCs subjected to oxidative stress.

### c-kit activation promotes migration of CPCs

**Fig 4:**
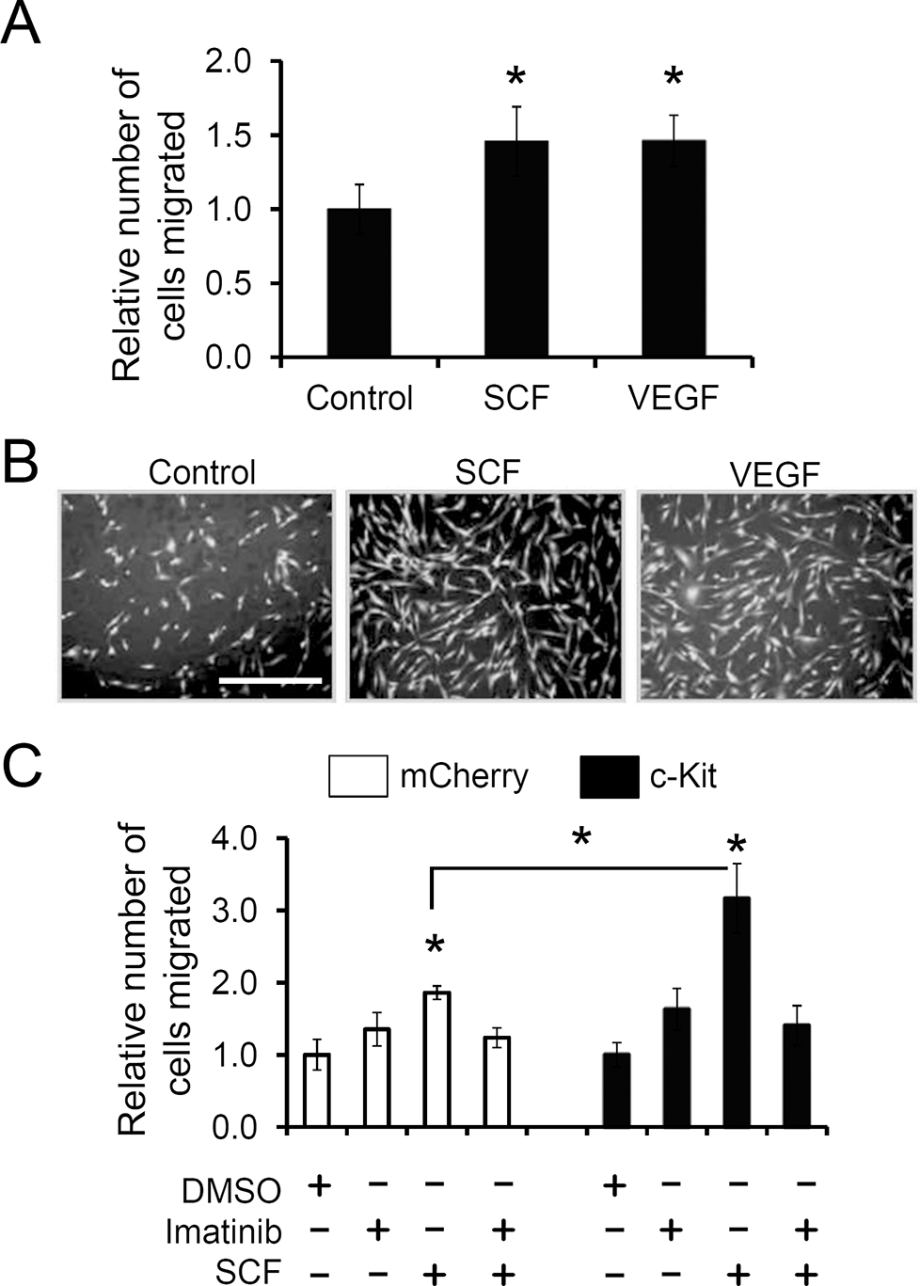
c-kit activation promotes migration of CPCs. Boyden chamber assay. Human c-kit+ CPCs were plated in the upper chamber, and the indicated growth factors were used as a chemoattractant in the lower chamber. After 24 hr, the migrated cells were fixed, stained with propidium iodide, and counted. A, Comparison of the pro-migratory effect of SCF and VEGF. B, Representative images showing the cells that migrated towards the indicated growth factors. C, CPCs transduced with lentivirus expressing mCherry (control) or c-kit were compared for their chemotaxis towards SCF in the presence (+) or absence (-) of 0.5 mM imatinib. Values presented are mean ± SEM. *, p<0.05.

For successful engraftment of the donor cells following transplantation, homing of the CPCs to the recipient myocardium and the injured area is required. SCF/c-kit signaling has been implicated in stimulating migration of various c-kit-expressing cell types [40, 49, 50]. Therefore, we sought to investigate whether SCF/c-kit signaling contributes to migration of CPCs. To test this, we implemented the Boyden chamber assay and used SCF as a chemo attractant. CPCs migrated towards the SCF gradient, and such chemotactic effect of SCF was comparable to that of VEGF (Fig 4A and 4B), which has previously been shown to promote migration of CPCs [51]. To further confirm this finding, we overexpressed c-kit (or mCherry as a control) in CPCs using lentivirus and performed the same migration assay. As expected, upon c-kit overexpression and SCF treatment, the number of migrated cells was further increased compared to the mCherry-expressing control CPCs (Fig 4C). Moreover, inhibition of c-kit with imatinib abolished the pro-migratory effect of SCF/c-kit signaling (Fig 4C), suggesting that the chemotactic effect of SCF on CPCs are indeed mediated through activation of the c-kit receptor.

### PI3K and MAPK pathways mediate the pro-growth and chemotactic effects of SCF/c-kit on CPCs

**Fig 5:**
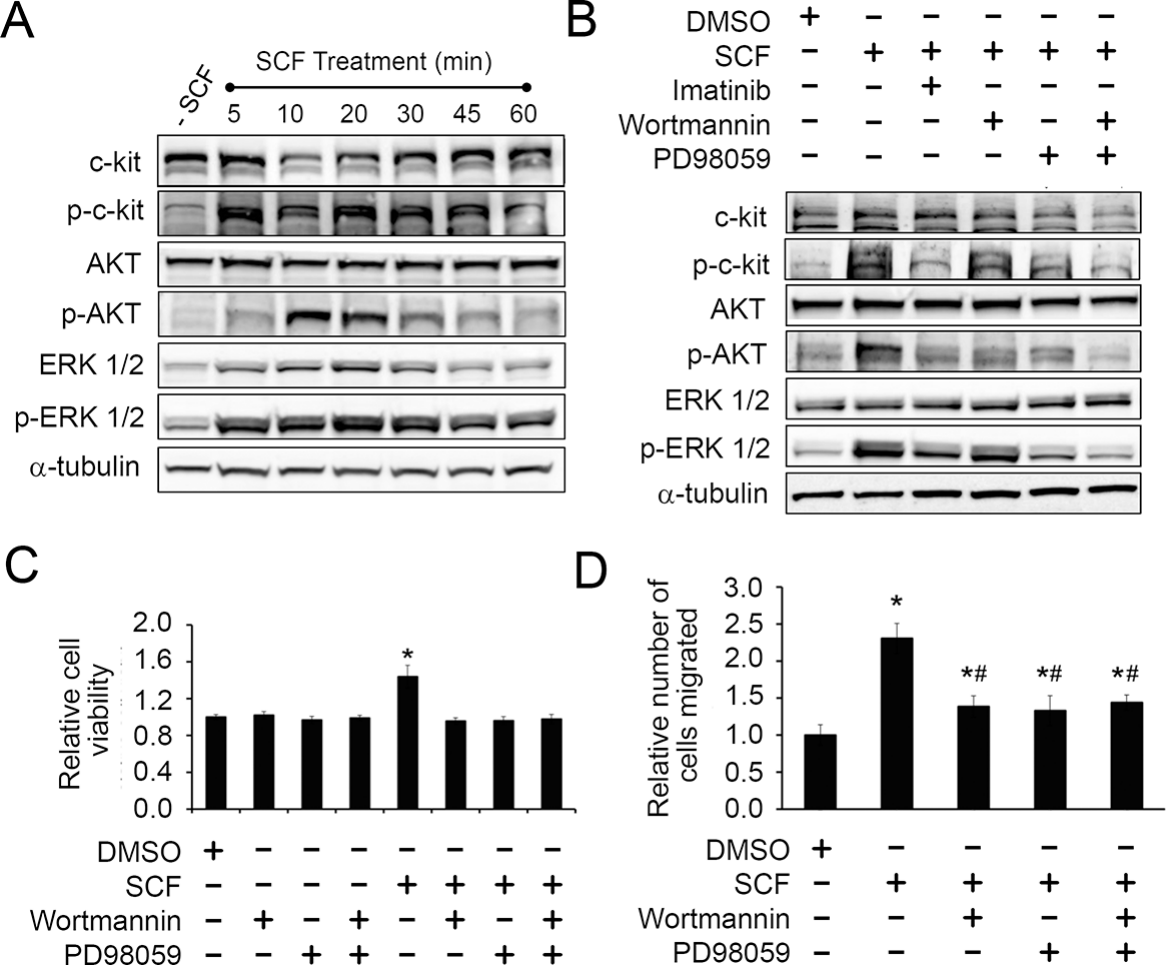
The effects of c-kit activation on CPCs are mediated via the PI3K-AKT and the MEK-ERK pathways. A, Human c-kit+ CPCs were either untreated (- SCF) or treated with 100 ng/ml SCF for the indicated duration. Levels of the indicated proteins at each time point were analyzed by Western blot. p-, phosphorylated (i.e., activated) form. B, CPCs were pre-treated with the indicated inhibitors for 2 hr followed by 20 min of SCF treatment. Levels of the indicated proteins were analyzed by Western blot. Imatinib, a c-kit inhibitor; Wortmannin, a PI3K inhibitor; and PD98059, a MEK inhibitor. C, Cell viability assay. CPCs were cultured in serum-free medium for 3 days in the presence (+) of absence (-) of SCF and the indicated inhibitors. Cell viability was assessed using PrestoBlue^TM^. D, Boyden chamber assay for cell migration. CPCs were plated in the transwells. After 24 hr, they were pre-treated with the inhibitors for 2 hr, and SCF was then added to the bottom chamber. Migrated cells were fixed, stained with propidium iodide, and counted. All values are normalized to the DMSO (vehicle) control. Values presented are mean ± SEM. *, p<0.05 compared to the DMSO control. #, p<0.05 compared to the SCF only treatment group.

Activation of c-kit leads to subsequent activation of different downstream pathways, including the PI3K-AKT [52, 53], p38MAPK [54, 55], MEK-ERK [56, 57], SFK [56], phospholipase C [58] and JAK/STAT [59] pathways. Among these, the PI3K-AKT and the MEK-ERK pathways represent the major pathway activated upon c-kit stimulation in a wide variety of cell types [29, 60]. Therefore, we tested if these two pathways also mediate the pro-survival and pro-migratory effects of c-kit activation in CPCs. First, we examined if these two pathways are subsequently activated upon c-kit activation. We treated CPCs with SCF for varying durations ranging from 5 to 60 minutes, and analyzed the changes in the phosphorylated (i.e., activated) AKT and phosphorylated ERK levels by Western blot. A significant increase in the level of phosphorylated AKT was observed within 10 min of SCF treatment, while the phosphorylated ERK levels increased as early as 5 min of SCF treatment (Fig 5A). These observations suggest that both the PI3K-AKT and MEK-ERK pathways are indeed activated upon c-kit activation in CPCs. Next, we investigated the contribution of each pathway to the biological effects of SCF/c-kit signaling on CPCs. For this, we utilized a PI3K inhibitor, Wortmannin, and a MEK inhibitor, PD98059. Western blot analysis confirmed that both inhibitors were effective in down-regulating their corresponding pathways, which was indicated by a significant reduction in the levels of activated AKT or activated ERK in cells co-treated with SCF and the inhibitors (Fig 5B). Interestingly, inhibition of the MEK-ERK pathway also inhibited the PI3K-AKT pathway but not vice versa (Fig 5B), suggesting that the MEK-ERK pathway operates upstream of the PI3K-AKT pathway. Pre-treating CPCs with these inhibitors (i.e., Wortmannin and PD98059) either individually or in combination abolished the pro-survival effect of SCF when the CPCs were cultured under serum starvation, while treating the cells with inhibitors alone did not affect their cell numbers (Fig 5C). Next, we tested if the pro-migratory effect of SCF is also dependent on PI3K-AKT and/or MEK-ERK pathways. While SCF alone stimulated cell migration (as shown earlier), pre-treatment of the cells with either one of the inhibitors prior to the SCF treatment prevented CPCs from migrating towards the SCF gradient (Fig 5D). These results indicate that the PI3K-AKT and the MEK-ERK pathways are the major pathways that regulate both pro-survival and -migratory effects of c-kit activation in human CPCs.

### Cardiac differentiation of CPCs are largely unaffected by c-kit activity

**Fig 6:**
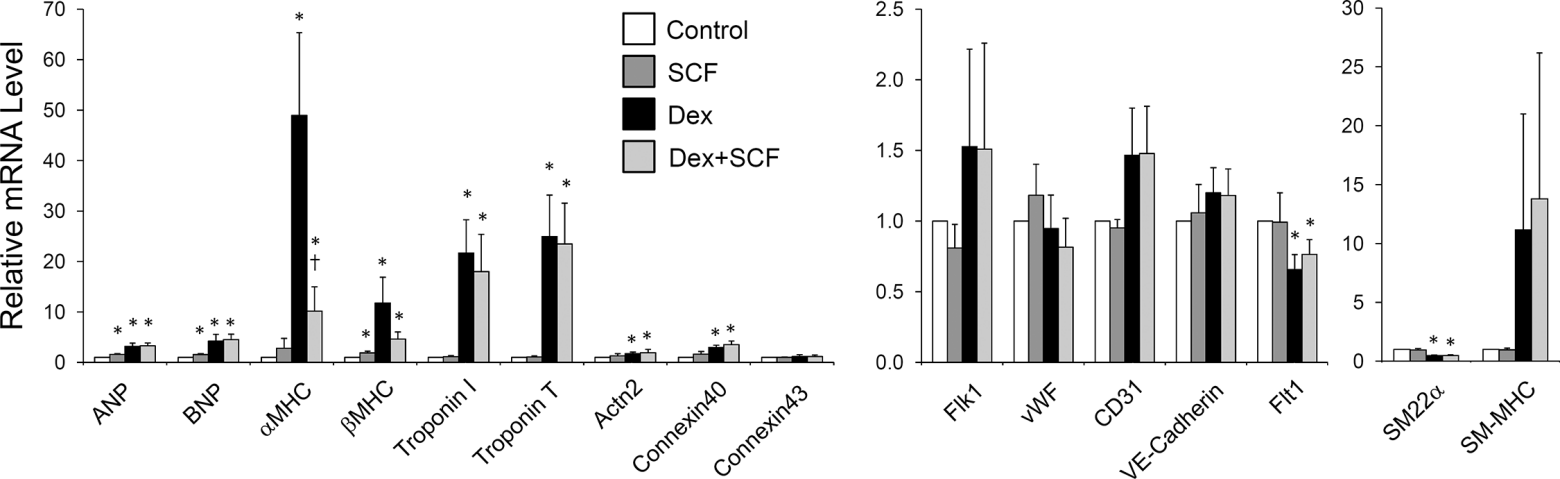
The effect of c-kit activation on cardiac differentiation of CPCs. Murine c-kit+ CPCs were induced to differentiate in dexamethasone-containing media as described in Materials and Methods. Cells in the first two groups were incubated in serum reduced media without dexamethasone and treated either with (SCF) or without (Control) SCF. The latter two groups were treated with 10 nM dexamethasone in the presence (Dex+SCF) or absence (Dex) of SCF. The mRNA levels of indicated cardiac markers were assessed by qPCR. *, p<0.05 compared to the Control. †, p<0.05 compared to the Dex only treatment group.

Next, we tested if c-kit activity has any significant effect on the ability of CPCs to differentiate into cardiac lineages. For this, we subjected murine c-kit+ CPCs to a dexamethasone-induced differentiation protocol and co-treated the cells with or without SCF. Dexamethasone was able to mostly induce markers of myocytes in CPCs (Fig 6). Although, compared to the dexamethasone only treatment group, there was a significant decrease in the α-MHC mRNA level following SCF treatment, SCF failed to alter the overall gene expression profile in differentiating CPCs (Fig 6). This suggests that the c-kit/SCF axis does not play a significant role in cardiac differentiation of CPCs.

## Discussion

Initial attempts to treat ischemic cardiomyopathy with administration of autologous c-kit+ CPCs have yielded promising results in both animals and humans, many unanswered questions remain regarding the use of these cells for cardiac repair, and many hurdles need to be overcome to establish the efficacy of this new therapy [61]. There is still a paucity of information regarding the biology and therapeutic capacity of c-kit+ CPCs. A better understanding of this cell population should allow us to better predict the behavior of these cells, overcome their limitations, and fully harness their inherent reparative activity.

The present study aimed at investigating the role of a cardiac stem/progenitor cell antigen, c-kit, in regulating the cellular properties of adult human cardiac c-kit+ CPCs. Our findings demonstrate, for the first time, that i) activation of c-kit leads to increased proliferation and survival and stimulates migration/chemotaxis in human CPCs, and ii) both the PI3K-AKT and MEK-ERK pathways are involved in mediating the salutary effects of SCF/c-kit signal in CPCs. These results imply that the SCF/c-kit signaling pathway in CPCs can be exploited to overcome one of the major problems in the current field of cardiac cell therapy: poor survival and engraftment of the transplanted cells.

For successful cell therapy of ischemic cardiomyopathy, the injected cells must find their ways into the host myocardium and to the site of injury. Hence, homing and migration of these CPCs after transplantation are critical cellular properties that determine their therapeutic and regenerative activities. However, this poses a major problem especially when the cells are delivered via the intracoronary route, as cells must transverse through the endothelial barrier to enter into the interstitial space and migrate towards the infarcted area. In a recent study, we have shown that > 60% of the injected CPCs are lost during the first 5 min of intracoronary injection [19], suggesting that the majority of them are immediately washed away by the coronary blood flow. Moreover, during the ensuing 24 hr, > 85% of the cells remaining at 5 min disappear [19]. Such rapid loss of the transplanted cells can be explained, at least in part, by inefficient homing and engraftment; this represents a major hurdle for CPC therapy. One of the findings of the present study is that activation of c-kit by its ligand SCF can stimulate migration/chemotaxis of human CPCs. This implies that augmentation of the SCF/c-kit signaling pathway could be an attractive means to enhance CPC homing and engraftment in the context of cardiac cell therapy.

The SCF/c-kit pathway is often activated and plays an important role during injury in various tissues [62–64]. For example, Sun and colleagues observed that SCF expression increases at the injury site following cryo-injury of the brain and functions as a chemoattractant to neural stem cells [62]. Interestingly, Myocardial ischemia-reperfusion injury also leads to increased expression of the kit ligand, SCF, specifically, in the injured segment, and this is accompanied by infiltration of c-kit+ cells into the peri-infarct zone [65], suggesting that c-kit-expressing progenitor cells from the bone marrow are attracted and supported by elevated production of SCF in the injured area [66]. It is well documented that following ischemic cardiac or vascular injury, c-kit+ lin- progenitors are mobilized from the bone marrow, recruited to the injury site, and serve cardioprotective roles [65–68]. The SCF/c-kit pathway is crucial for mobilization and recruitment of bone marrow-derived c-kit+ progenitor cells (e.g., endothelial progenitor cells) in response to cardiac injury. Following cardiac injury, the membrane-bound form of SCF is cleaved by MMP-9 and released within the bone marrow, which leads to mobilization of bone marrow progenitor cells [65, 69]. Accordingly, the mobilization of bone marrow-derived progenitor cells and their recruitment to the injured heart are severely compromised in the c-kit mutant (*Kit* ^*W*^ */Kit* ^*W-v*^) mouse [65], demonstrating that activation of c-kit is indeed necessary for homing of the bone marrow-derived progenitor cells. SCF not only works as a chemotactic signal to recruit c-kit+ bone marrow progenitor cells but also promotes adhesion of the recruited cells to the injury site. For instance, activation of c-kit by SCF enhances the adhesion of c-kit+ bone marrow cells to fibronectin as well as to the activated vascular smooth muscle cells following vascular injury [67]. These observations suggest that SCF/c-kit signaling not only provides a chemotactic signal to recruit c-kit+ progenitors to the injury site, but also facilitates their engraftment. Corroborating this, Lutz and co-workers showed that local intramyocardial injection of SCF improves myocardial homing of systemically delivered c-kit+ bone marrow-derived stem cells [70]. Although we have not examined whether activation of SCF/c-kit signaling can promote adhesion of CPCs either to endothelium or injured myocardium, it is tempting to speculate that SCF/c-kit signaling also contributes to adhesion and engraftment of the transplanted c-kit+ CPCs to the injured regions in the setting of regenerative cardiac cell therapy.

The functional importance of SCF/c-kit signaling in regulating c-kit+ CPCs and cardiac regeneration is further underscored by a recent study that demonstrated the benefits of SCF therapy. A study by Yaniz-Galende and colleagues showed that adenovirus-mediated gene delivery of the membrane-bound form of SCF to the myocardium can provide long-term improvement in cardiac structure and function in an animal model of myocardial infarction (MI) [71]. The SCF gene therapy was accompanied by a marked increase in the number of cardiac c-kit+ cells at 1 week post-MI. Moreover, there was a concomitant increase in the percentage of c-kit+ cells that expressed early cardiac commitment markers such as Gata4, Nkx2.5, and Mef2. Interestingly, this activation of cardiac c-kit+ population was followed by a significant induction of cardiomyocyte regeneration in the SCF gene therapy group. These observations suggest that activation of the c-kit receptor by exogenous SCF leads to recruitment as well as proliferation and differentiation of the cardiac c-kit+ progenitor cell population, ultimately contributing to cardiac repair and regeneration. A recent study by Sanada et al. demonstrated that resident c-kit+ CPCs in their quiescent state can be activated *in situ* by intramyocardial injection of SCF and potentiate the regenerative response in the aging murine heart [72]. The authors observed that resident c-kit+ CPCs in hypoxic microenvironments remain quiescent and that there is an age-related increase in the number of hypoxic niches harboring quiescent CPCs. When stimulated with intramyocardial injection of SCF, c-kit+ CPCs were induced to proliferate. Notably, activation of c-kit+ CPCs was followed by appearance of newly formed, BrdU-labeled myocytes and reversal of age-related decrements in cardiac anatomy and function [72]. These studies highlight the importance of the SCF/c-kit signaling in the regulation of c-kit+ CPC activity and the potential utility of therapeutic intervention targeting this pathway.

Our present findings that c-kit activation elicits pro–growth and–migratory signals in human c-kit+ CPCs are consistent with these previous observations. As such, augmentation of the inherent c-kit activity in exogenous CPCs appears a viable option to further improve the outcome of CPC-based cardiac cell therapy. Additional studies using animal models of myocardial ischemia-reperfusion are required to test if such strategy is able to enhance post-transplantation survival and engraftment of the cells and ultimately augment the efficacy of cell therapy. Also, it will be of interest to study how differences in the percentage of c-kit-expressing cells or in the level of c-kit expression among patients translate into differences in the therapeutic efficacy of individual CPC populations.
